# A phase II study of sorafenib (BAY 43–9006) in recurrent diffuse large B cell lymphoma: an eastern cooperative oncology group study (E1404)

**DOI:** 10.1186/1756-8722-6-46

**Published:** 2013-07-05

**Authors:** Daniel R Greenwald, Hailun Li, Selina M Luger, Ronald S Go, David King, Taral Patel, Randy D Gascoyne, Jill Kolesar, Brad S Kahl, Sandra Horning

**Affiliations:** 1 Stanford University, Stanford, CA, USA; 2 Dana Farber Cancer Institute, Boston, Massachusetts, USA; 3 University of Pennsylvania, Philadelphia, PA, USA; 4 Gundersen Lutheran Health System, La Crosse, WI, USA; 5 Health One Mercy Hospital, Coon Rapids, MN, USA; 6 Columbus CCOP, Columbus, OH, USA; 7 British Columbia Cancer Agency, Vancouver, Canada; 8 University of Wisconsin, Madison, WI, USA; 9 Roche-Genentech, South San Francisco, CA, USA; 10 Cancer Center of Santa Barbara, 540 West Pueblo St, Santa Barbara, CA 93105, USA

**Keywords:** NHL, Diffuse large B cell lymphoma, MAP kinase signaling, Sorafenib

## Abstract

Patients with diffuse large B cell lymphoma (DLBCL) who are not candidates for or recur after autologous stem cell transplant have a poor overall prognosis. We conducted a phase II study of sorafenib (formerly BAY 43–9006) in the treatment of relapsed DLBCL. Fourteen patients were enrolled and assessed for response. Median number of cycles administered was 3 (range, 1–12). Common grade 3 toxicities included fatigue (29%), rash/desquamation (21%) and diarrhea (14%). One complete response (CR) was observed (the 14th patient enrolled). Response rate was 7% (90% CI, 0.4 – 30%). Duration of response was 6 months. Median progression-free survival (PFS) was 2 months (90% CI, 1 – 5 months). Median overall survival (OS) was 9 months (90% CI, 5 – 16 months). Although sorafenib has demonstrated activity in solid malignancies it demonstrated low single agent activity in treatment of DLBCL.

## Introduction

The non-Hodgkin lymphomas remain among the most treatable forms of cancer. In spite of success with present chemotherapy and antibody-based regimens, a large subset of patients will recur after primary and secondary treatment. While effective in many cases, chemotherapy based treatment carries risks of substantial short and long-term toxicity. Patients who relapse after standard therapy may be eligible for high dose therapy with stem cell transplant. This approach cures fewer than half of patients with relapsed disease [1,2] and many patients are not eligible on the basis of age or other comorbidities. More effective, less toxic therapies are needed.

*Ras* oncogene activation plays an instrumental role in carcinogenesis of several human tumor types including several hematologic malignancies [3,4]. The *Ras/Raf*/MEK/ERK kinase pathway may play a role in pathogenesis, tumor signaling, apoptosis susceptibility, and treatment resistance observed in several *in vitro* lymphoma models [5-10]. Vascular endothelial growth factor also contributes to lymphoma formation and progression and is an active area of therapeutic investigation [11-14] Sorafenib blocks tumor angiogenesis by downstream inhibition of VEGFR-2/PDGFR-ß.

Sorafenib is a bis-aryl urea which inhibits the VEGFR-2/PDGFR-ß and *Ras/Raf*/MEK/ERK signaling pathways [15-17]. Sorafenib is approved by the United States Food and Drug Administration for the treatment of renal cell carcinoma and hepatocellular carcinoma [18,19]. Based on the preclinical activity and toxicity profile we performed a phase II clinical trial of sorafenib in patients with relapsed DLBCL who failed or were not candidates for autologous stem cell transplant.

## Materials and methods

We conducted a two-stage phase II study to assess safety and activity of sorafenib in patients with relapsed aggressive DLBCL. Response assessment was based upon the criteria from the International Workshop to Standardize Criteria for non-Hodgkin Lymphoma [20]. The study was conducted through the Eastern Cooperative Group and was approved by the respective Institutional Review Boards. Patients with *de novo* or transformed DLBCL were eligible if they had previously received therapy with curative intent and had relapsed greater than 2 months after their last treatment. Patients were required to have progressed after or be ineligible for autologous stem cell transplant. Eligibility criteria included age greater than 18 years old, ECOG performance status (PS) of 0–1, measurable disease by computed tomography, absolute neutrophil count count ≥ 1,000/mm^3^, platelet count ≥ 75,000/mm^3^, normal serum creatinine, total bilirubin ≤ 2.0 times institutional upper limit of normal, AST ≤ 2.5 × institutional upper limit of normal, ALT ≤ 2.5 times institutional upper limit of normal, and normal PT/INR.

Patients received sorafenib at a dose of 400 mg PO BID continuously in 28-day cycles. Patients who showed no disease progression at the end of cycle 2 were to receive an additional 4 cycles (for a total of 6 cycles) of sorafenib. Patients who were responding or stable at the end of cycle 6 were to continue to receive 28-day cycles of sorafenib until progressive disease or excessive toxicity.

Patients were instructed to take the tablets every 12 hours with an 8 oz. glass of water, with or without food. If sorafenib was taken with meals, patients were instructed to take sorafenib with a moderate to low-fat meal. To track compliance, patients were required to complete a pill calendar. Adverse events reporting requirements and appropriate dose modifications in case of toxicities were described in the protocol. Patients were restaged for response after 2 and 6 cycles using the International Workshop Criteria. Patients who progressed or had unacceptable toxicity at any time discontinued therapy. Patients with stable disease after 6 cycles continued treatment at physician’s discretion. Responding patients were to continue on medication.

### Statistical design and method

The study used a two-stage Simon design [21] to assess the clinical efficacy of sorafenib in patients with relapsed DLBCL. A total of 37 eligible patients were required to test the null hypothesis that the true response rate for this regimen is at most 5% versus the alternative hypothesis that the true overall response rate is 20% or greater. In first stage, 13 patients (12 eligible) were to be entered. If at least 1 response was observed among the 12 eligible patients, an additional 28 patients (25 eligible) were to be entered. Treatment would be considered promising with at least 4 responders out of the 37 eligible patients.

Descriptive statistics were used to characterize patients at study entry. Toxicities were assessed using the *NCI Common Terminology Criteria for Adverse Events (CTCAE) Version 3.0*. Exact binomial confidence intervals were used to describe response rate. Progression-free survival (PFS) was defined as the time from study entry to progression or death. Overall survival (OS) was defined as the time from study entry until death from any cause. PFS and OS were estimated using the Kaplan-Meier method.

## Results

### Administrative information

The study was activated on October 25, 2005, and was suspended on December 15, 2006 for pre-planned response evaluations after accruing 14 patients. No response was observed in the first 12 eligible patients. Patient #14 was enrolled prior to notice of accrual suspension for planned response assessment. Although 1 complete response (CR) was later confirmed, this patient (the 14th patient enrolled) was not among the first 12 eligible patients. Based on the initial trial design of lack of response activity for the first 13 patients, the study was terminated on September 25, 2007. The median follow-up was 36 months. Eight ECOG institutions contributed patients to the study. All 14 patients were eligible. Central pathology review was done for 11 (79%) and 3 cases were unavailable for central review.

### Patient characteristics

Patient characteristics at study entry are summarized in Table 1. Patients ranged in age from 38 to 88 years, with a median of 69.5 years. All patients were white, and 64% were males. Seven (50%) patients had ECOG PS of 0, and the other 7 (50%) had PS of 1. Eight (57%) had no extra-lymphatic sites involved, 1 (7%) had one site, 3 (21%) had two sites, and 2 (14%) had more than two sites. One patient (7%) had bone marrow involvement, 6 (43%) had elevated LDH, and 5 (36%) had lymph node or aggregate with a diameter > 5 cm. None of the patients had B symptoms present or mediastinal mass. All patients had prior chemotherapy, 4 (29%) had prior radiation therapy, 1 (7%) had prior surgery with therapeutic intent, 3 (21%) had prior bone marrow transplant (autologous), and 1 (7%) had radioimmunotherapy (Zevalin).

**Table 1:** Baseline characteristics

	**N**	**%**
Total number of patients	14	
Age		
Median (Range)	69.5 (38–88)	
Gender		
Male	9	64
Female	5	36
Race		
White	14	100
Performance Status (PS)		
0	7	50
1	7	50
Extra-lymphatic sites involved		
0	8	57
1	1	7
2	3	21
>2	2	14
Elevated LDH	6	43
Bone Marrow Involvement	1	7
B symptom	0	0
Mediastinal mass	0	0
Lymph node or aggregate with a diameter >5 cm	5	36
Prior Treatment		
Prior Chemotherapy	14	100
Prior Immunotherapy	4	29
Prior Radiation Therapy	4	29
Prior Surgery (with therapeutic intent)	1	7
Prior Bone Marrow Transplant*	3	21
Other Prior Therapy**	1	7

* All three are autologous BMT.

** Patient 14002: ZEVALIN® (ibritumomab tiuxetan).

### Treatment

All 14 patients started protocol treatment. Table 2 shows the number of cycles administered and reasons for discontinuing treatments. The median number of cycles administered was 3 (range, 1–12). Seven patients (50%) went off treatment due to disease progression, with only one receiving more than six cycles of therapy. Three (21%) went off treatment due to adverse events during cycle 1. Two patients withdrew after cycle 2 and cycle 3, one started alternative therapy (external beam radiation) after cycle 3, and one was taken off the study after cycle 6 by treating physician.

**Table 2:** Cycles received and off treatment reasons

	**Cycles**							
**Reasons off treatment**	**1**	**2**	**3**	**5**	**6**	**12**	**Total**	
	**(n)**	**(n)**	**(n)**	**(n)**	**(n)**	**(n)**	**(n)**	**(%)**
Disease progression	1	1	1	1	2	1	7	50
Adverse events	3						3	21
Patient withdrawal		1	1				2	14
Alternative therapy			1				1	7
Other*					1		1	7
Total	4	2	3	1	3	1	14	100

* 14009: MD decision to withdraw due to lack of efficacy and side effects.

### Toxicity

Table 3 summarizes toxicities classified at least possibly treatment related. There were no treatment-related deaths. Grade 4 toxicities included one thrombocytopenia (7%) and one fatigue (7%). Common grade 3 toxicities were fatigue (4 cases, 29%), rash/desquamation (3 cases, 21%) and diarrhea (2 cases, 14%).

**Table 3:** Treatment related toxicities

	**Arm A (N = 14)**			
	**Grade**			
	**1,2**	**3**	**4**	**5**
	**(n)**	**(n)**	**(n)**	**(n)**
Allergic reaction	1	-	-	-
Hemoglobin	5	-	-	-
Leukocytes	-	1	-	-
Neutrophils	1	1	-	-
Platelets	3	1	1	-
Hypertension	6	1	-	-
Fatigue	7	4	1	-
Weight loss	1	-	-	-
Flushing	1	-	-	-
Alopecia	1	-	-	-
Pruritus/itching	1	-	-	-
Rash/desquamation	5	3	-	-
Hand-foot reaction	3	1	-	-
Skin-other	1	-	-	-
Anorexia	3	-	-	-
Constipation	2	-	-	-
Dehydration	-	1	-	-
Diarrhea w/o prior colostomy	6	2	-	-
Flatulence	1	-	-	-
Dyspepsia	3	-	-	-
Muco/stomatitis (symptom) oral cavity	2	-	-	-
Nausea	1	1	-	-
GI-other	1	-	-	-
Infection Gr0-2 neut, urinary tract	1	-	-	-
Infection w/ unk ANC upper airway NOS	1	-	-	-
ALT, SGPT	4	-	-	-
AST, SGOT	5	-	-	-
Bilirubin	3	-	-	-
Hyponatremia	-	1	-	-
Nonneuropathic generalized weakness	-	1	-	-
Musculoskeletal/soft tissue-other	1	-	-	-
Dizziness	1	1	-	-
Neuropathy-sensory	2	-	-	-
Extremity-limb, pain	1	-	-	-
Head/headache	1	-	-	-
Joint, pain	1	1	-	-
Muscle, pain	2	-	-	-
Dyspnea	1	-	-	-
Worst degree	5	7	2	-

### Response

Table 4 shows the best overall response. One patient (14014) had complete response (CR) at post cycle 6 disease assessment. This was the only response. Response rate was 7% (90% CI, 0.4 – 30%) with a duration 6 months. The patient received a total of 12 cycles of protocol therapy before disease progression. Central pathology review confirmed unclassifiable B-cell lymphoma for this patient.

**Table 4:** Best overall response

**Response**	**N**	**%**
Complete Response (CR)	1	7
Stable Disease (SD)	5	36
Progressive Disease (PD)	7	50
Unevaluable *	1	7

* Patient 14012 was unevaluable because he was taken off study after receiving only one cycle of protocol therapy due to adverse events, and never had follow-up disease evaluations.

Five patients (36%) had stable disease and 7 (50%) had progression as their best overall response. One patient (14012) was not evaluable for response because he was taken off study due to toxicities after receiving only 1 cycle of protocol therapy and never had follow-up disease evaluations.

### Progression-free survival

**Figure 1:**
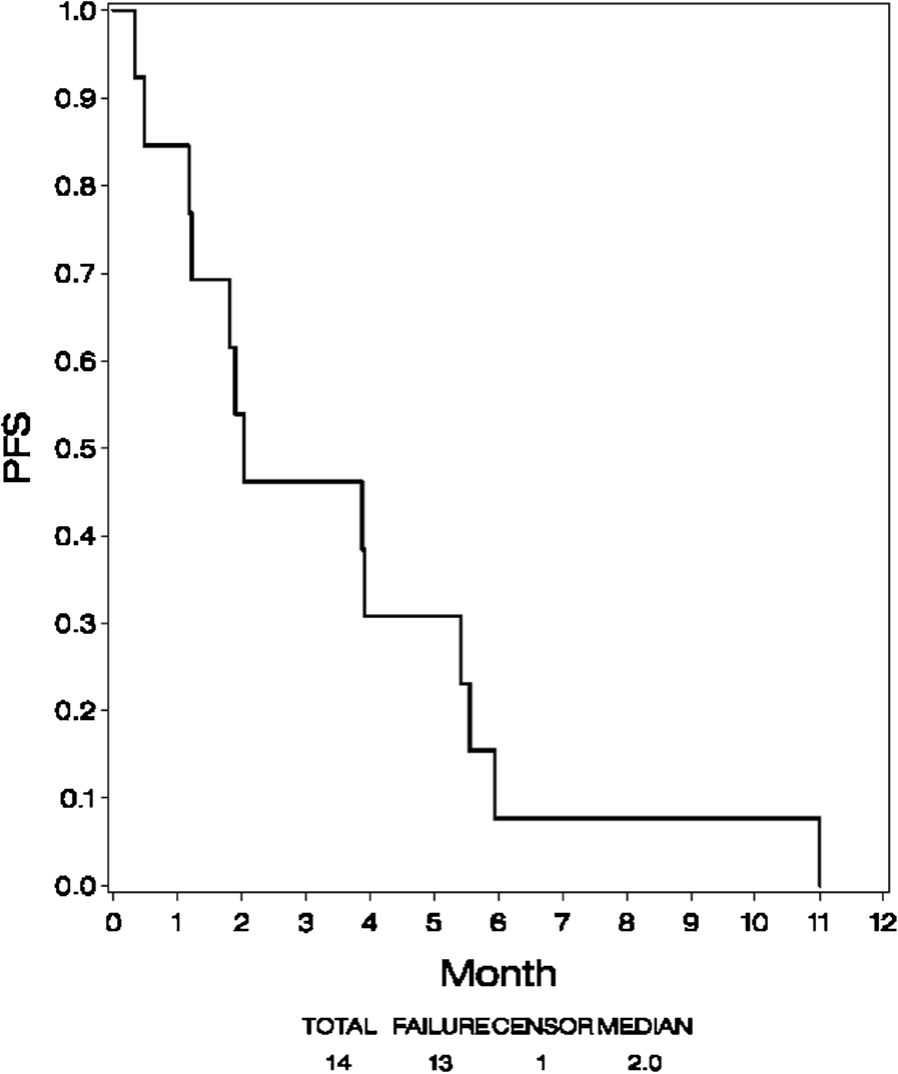
Progression-free survival (PFS).

Figure 1 shows PFS. Thirteen patients (93%) had documented progression. One patient never had follow-up disease evaluations, and therefore PFS was censored at time zero. Median PFS was 2 months (90% CI, 1 – 5 months).

### Overall survival

**Figure 2:**
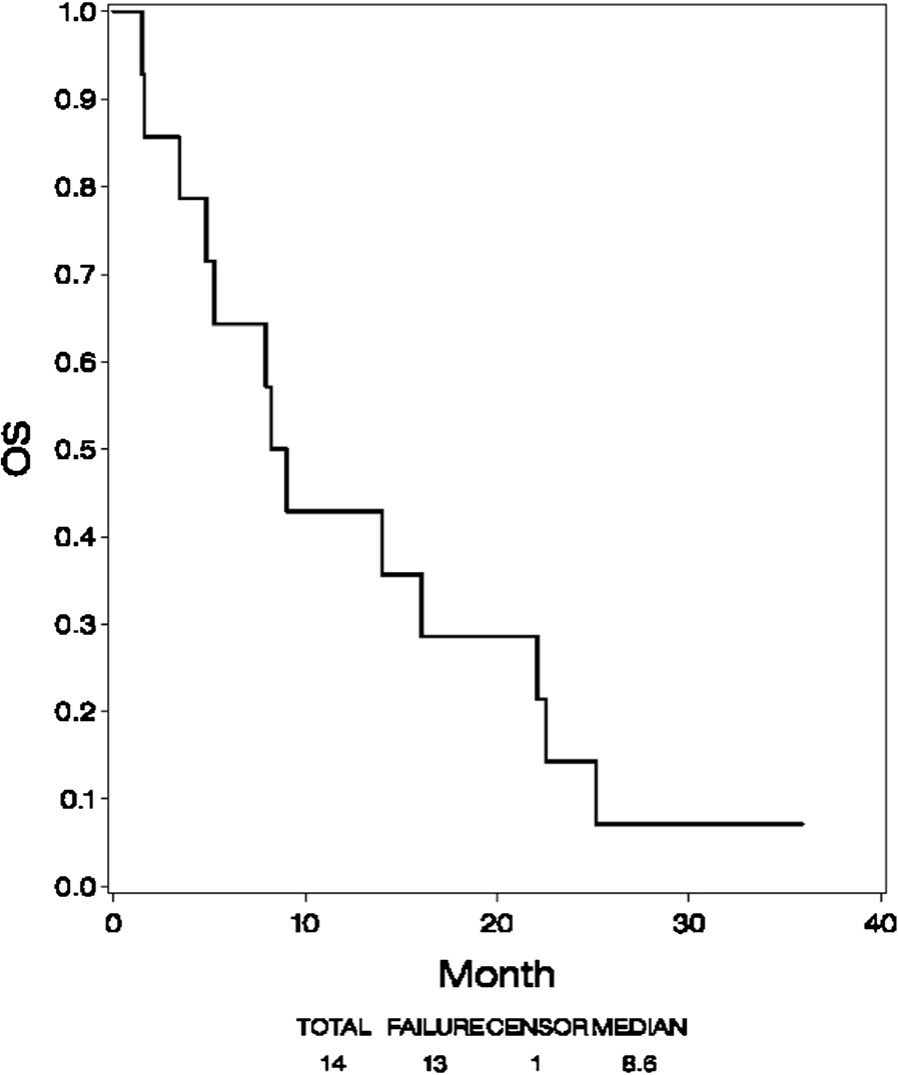
Overall survival (OS).

Figure 2 shows OS. Thirteen patients (93%) have died. Median survival was 9 months (90% CI, 5 – 16 months).

## Discussion

Sorafenib was reasonably well tolerated in pretreated patients with relapsed DLBCL. The toxicity profile was similar to that described in other disease trials with this agent [22]. One episode of grade 4 thrombocytopenia and one episode of grade 4 fatigue were observed. We demonstrated one complete response in a patient who subsequently progressed. Based on the analysis of the initial stage we did not meet our predefined primary end point of a 20% confirmed response rate as indicative for additional study of the drug. Although cytostatic effects of targeted agents have demonstrated improvements in PFS and OS through disease stability in other malignancies, the majority of the patients in this study succumbed to progressive disease. Other groups have evaluated the clinical activity of sorafenib and sunitinib in treatment of relapsed diffuse large B-cell lymphoma with reported overall response rates of 13 and 0% respectively [23,24]. When compared with previous results of agents undergoing evaluation for treatment of relapsed DLBCL, the results in this phase II trial showed less activity compared with agents considered to have clinically meaningful therapeutic effect including lenolidomide [25]. Therapies which target the B cell receptor pathway appear to demonstrate greater activity and are undergoing active investigation [26]. This was a small exploratory study intended to assess safety and activity of sorefenib in this population. It has not been tested in combination with standard therapy and it is unknown whether it might potentiate or enhance toxicity of standard therapy. Such combinations could be considered for exploration if additional preclinical data were supportive. On the basis of poor therapeutic efficacy observed in this trial additional targeted therapies should be explored.

## Competing interest

HL, SL, RG, DK, TP, RG, JK, BK – no current relevant competing financial interests. SH – current employment with Genentech/Roche.

## Authors’ contributions

DG and HL authored the manuscript. DG, SH, RG, HL, JK participated in conception and design of the study. HL perform the statistical analysis and preparation of figures. SL, RG, DK, TP contributed to trial implementation and data review. BK provided supervision of the research group and contributed to manuscript preparation and editing. RSG, RG participated in editing and preparation of the manuscript. RG supervised central pathology review. All authors read and approved the final manuscript.
